# Theoretical Investigation of Azobenzene-Based Photochromic Dyes for Dye-Sensitized Solar Cells

**DOI:** 10.3390/nano10050914

**Published:** 2020-05-09

**Authors:** Md Al Mamunur Rashid, Dini Hayati, Kyungwon Kwak, Jongin Hong

**Affiliations:** 1 Department of Chemistry, Chung-Ang University, Seoul 06974, Korea; ndcmamun@korea.ac.kr (M.A.M.R.); dinihayati300194@gmail.com (D.H.); 2 Center for Molecular Spectroscopy and Dynamics, Institute for Basic Science (IBS) & Department of Chemistry, Korea University, Seoul 02841, Korea

**Keywords:** dye-sensitized solar cells, azobenzene, density functional theory

## Abstract

Two donor-π-spacer-acceptor (D-π-A) organic dyes were designed as photochromic dyes with the same π-spacer and acceptor but different donors, based on their electron-donating strength. Various structural, electronic, and optical properties, chemical reactivity parameters, and certain crucial factors that affect short-circuit current density (*J_sc_*) and open circuit voltage (*V_oc_*) were investigated computationally using density functional theory and time-dependent density functional theory. The *trans*-*cis* isomerization of these azobenzene-based dyes and its effect on their properties was studied in detail. Furthermore, the dye-(TiO_2_)_9_ anatase nanoparticle system was simulated to understand the electronic structure of the interface. Based on the results, we justified how the *trans*-*cis* isomerization and different donor groups influence the physical properties as well as the photovoltaic performance of the resultant dye-sensitized solar cells (DSSCs). These theoretical calculations can be used for the rapid screening of promising dyes and their optimization for photochromic DSSCs.

## 1. Introduction

To meet the ever-increasing global energy demands, the utilization of solar energy—a clean, renewable, and naturally abundant energy resource—has attracted considerable attention in recent decades. Accordingly, photovoltaic devices (or solar cells) have been extensively developed to meet this energy demand. Dye-sensitized solar cells (DSSCs) have been widely investigated as a promising candidate for low-cost photovoltaic cells in the past two decades because of their distinctive features, including shape flexibility, transparency, better performance under prolonged low-light conditions, thermal dual stress, different solar incident angles, easy material synthesis, low weight, and cost-effectiveness. Moreover, new functional materials have been designed to increase the solar-to-electrical energy conversion efficiency of DSSCs [1,2]. In the public sector, DSSCs are used in flat and curved building skins for building-integrated photovoltaics because of their transparency and aesthetic value. Although numerous studies have been conducted based on device physics, material innovation, and commercialization to achieve high performance and long-term fidelity of DSSCs [3], they are still deficient in various aspects.

The photosensitizer is the core of a DSSC that absorbs solar radiation over a broad spectral range. Moreover, it contains functional groups, which aid in adsorption on the TiO_2_ surface and injection of electrons into the conduction band (CB) of TiO_2_ after solar light excitation. Organic dyes are attracting increased attention not only as alternative photosensitizers, but also as promising photofunctional materials for optical devices and photovoltaic cells because of their low cost, environment friendliness, and high molecular extinction coefficients [4]. Metal-free organic dyes, which commonly feature a push-pull architecture like dipolar donor–π-bridge–acceptor (D–π–A) frameworks, are being studied for use in DSSCs more than Ru-based dyes. This is because metal-free organic dyes have attractive attributes, such as efficient intramolecular charge transfer (ICT), a wider variety of structural designs, easy fabrication, raw material abundance, various synthetic protocols, good flexibility for molecular tailoring, tunable spectral properties, high efficiency, cost-effectiveness, and applicability as organic optoelectronic materials [5,6]; consequently, their commercial application is promising. Because of these features, recent research has focused on designing new metal-free organic dyes to further improve the performance of DSSCs. In the D–π–A structure, the donor unit plays an important role in not only tuning and modifying the absorption spectra but also controlling the molecular energy levels and intramolecular charge separation. Thus, several studies have been conducted to investigate the effect of changing the donor units on the absorption characteristics of the dyes and DSSC performance [7,8]. Although triphenylamine, dialkylamine, and diphenylamine moieties are commonly used as electron donors [4,9], only a few studies have systematically investigated the molecular origin of the DSSC performance modulated by these donor groups.

Azobenzene dyes are organic compounds that contain the photoreactive -N=N- group, which undergoes reversible *trans*-*cis*-*trans* isomerization when irradiated by sunlight. Therefore, these compounds are used in photoresponsive material systems as phototriggers [10]. Azobenzene photochemistry has also been observed in numerous constricted and/or interfacial environments, such as molecular or liquid crystals for molecular level photoswitching, or embedded within cyclodextrins, polymers, and metal-organic frameworks [11,12]. Recently, D–π–A-type azobenzene derivatives have generated considerable interest because of the presence of both, electron-donating and electron-accepting groups, on the π-conjugated system of the azo chromophore. Several studies have been conducted on conjugated π-spacers, such as acetylene, vinyl, and phenyl [8,13]. However, in metal-free organic dyes, the effect of using azobenzene as a π-spacer in the D–π–A structure has not been widely studied; examples of the effective inclusion of azobenzene dyes into DSSCs are rare, and the correlation between the molecular arrangement of these dyes and DSSC properties has not been studied extensively [9].

Quantum chemical methods have been employed in recent decades as a sustainable approach for elucidating the relationship between molecular geometries and dye characteristics, thus offering a reliable theoretical platform for the rapid screening of efficient dyes prior to expensive and time-consuming syntheses. Density functional theory (DFT) and time-dependent density functional theory (TDDFT) have been extensively used to investigate the electronic and optical properties of virtual photosensitizers in the ground and excited states for the development of DSSCs [14,15]. Therefore, the theoretical predictions based on DFT calculations are promising, as they correlate well with the experimental data on DSSCs [16]. Numerous research groups have successfully calculated the photoelectric properties of organic dyes using quantum chemical methods. Donor modifications can improve the light-harvesting efficiency (LHE) and electron injection ability, which contribute to the solar cell efficiency [8]. The use of a bulky donor moiety leads to a high open circuit voltage, longer electron lifetime, and slower back-transfer of electrons, resulting in higher photovoltaic performance [17]. The role of donor moieties in the photoinjection mechanism has also been investigated for a series of D–π–A-structured dyes adsorbed on a (TiO_2_)_15_ anatase cluster in the DFT framework using various functionals [18]. Novir et al. investigated the properties of numerous azobenzene-based dyes with different electron-donating groups and reported that the donor groups did not have any significant effect on their optical properties, such as LHE and exciton binding energy [19].

In this study, two photochromic azobenzene-based dyes were selected as sensitizers to investigate the various properties of DSSCs to determine the relationship between the molecular structure and photoelectric properties using reliable quantum chemical calculation methods. The objective of this study was to understand the effect of different donor groups (dimethylamine and diphenylamine) on the photophysical properties of the two azo dyes and the photovoltaic performances of the resultant DSSCs. For in-depth analysis via DFT and TDDFT, the structural, electronic, optical properties, including chemical reactivity parameters and some crucial factor relating to short circuit current density (*J_SC_*) and open circuit voltage (*V_OC_*) of the two dyes were determined after their adsorption on a TiO_2_ surface. The elaborate DFT analyses presented herein can provide a better understanding of the photoelectrical properties of the two azo dyes for photochromic DSSCs.

## 2. Methods

The ground-state geometries of all the dyes before and after binding onto the TiO_2_ surface were fully optimized using *N*,*N*-dimethylformamide (DMF) solvent (ε = 37.5) without symmetry constriction. Frequency calculations were performed to confirm that all the optimized geometries were stationary minima points. The calculations were carried out using DFT at the B3LYP level with the 6-311G(d,p) basis set for C, H, O, and N atoms and the LANL2DZ basis set for the Ti atom [20], considering the relativistic effect of heavy atoms. The excitation energies, oscillator strengths, and UV-Visible absorption spectra of all the dyes before and after binding to TiO_2_ in the DMF solvent were simulated using TDDFT with CAM-B3LYP [21] functionals and the 6-311++G(d,p) basis set for non-metal atoms, and the LANL2DZ basis set for the Ti atom on the basis of the optimized ground-state geometries. The effective core potential (ECP) for sixty valence electrons of the dyes adsorbed on the TiO_2_ surface was applied for the DFT and TDDFT calculations. The conductor-like polarized continuum model (C-PCM) method [22] was applied within the self-consistent reaction field theory to simulate the solvent effects throughout the study. Natural bond orbital (NBO) analysis was performed by calculating the orbital populations for the ground state and excited state using the NBO 5.0 program [23]. All calculations were performed using the Gaussian 16 package [24].

## 3. Results and Discussion

### 3.1. Isolated Dyes and Dye/TiO_2_ Complexes

**Figure 1:**
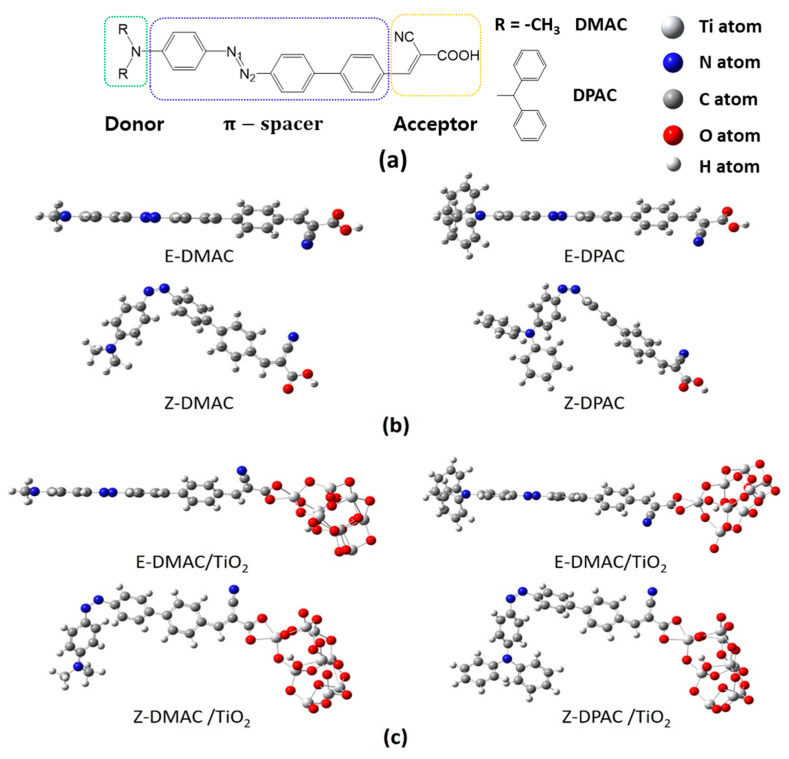
(**a**) Molecular structure of the dyes, and optimized geometries for *trans* (E) and *cis* (Z) structures of 2-cyano-3-(4′-(4-(dimethylamino)phenyl)diazenyl)-[1,1′-biphenyl]-4-yl)acrylic acid (DMAC) and 2-cyano-3-(4′-(4-(diphenylamino)phenyl)diazenyl)-[1,1′-biphenyl]-4-yl)acrylic acid (DPAC) as (**b**) isolated dyes and (**c**) dye/TiO_2_ complexes. The titanium, nitrogen, carbon, oxygen, and hydrogen atoms are shown in the legend.

In this study, two D–π–A organic dyes were designed containing two electron-donating moieties, namely, dimethylamine and diphenylamine, an azobenzene-benzene moiety as the π-spacer, and cyanoacrylic acid as the anchoring group, as shown in Figure 1. The azo group, which showed reversible *cis*-*trans* photoisomerization and allowed geometrical change of the π-conjugation backbone under light and heat, led to the *trans* and *cis* structures of the two studied dyes (Figure 1b). In this study, the *trans* structures are named *E*-DMAC and *E*-DPAC and the *cis* structures are named *Z*-DMAC and *Z*-DPAC. Here, DMAC and DPAC contained methyl and phenyl moieties in their donor moieties, respectively. To provide more realistic information about the dye adsorption on the semiconductor surface in terms of electronic structure and optical properties, the dyes adsorbed on the TiO_2_ surface were also studied and are referred to as dye/TiO_2_ in this study. Figure 1c shows the optimized structures of the dye/TiO_2_ complexes for both dyes. In the dye/TiO_2_ complexes, the adsorption of dyes through carboxylic acid can occur via either physisorption or chemisorption. The carboxylic acid can bind to the TiO_2_ surface by several anchoring modes, such as monodentate bridging, bidentate bridging, and bidentate chelating [25,26]. Because of the controversies surrounding the exact anchoring modes for the binding of dyes on TiO_2_ nanoparticles, the studied dyes were optimized considering all the three anchoring modes, and the findings revealed that the bidentate chelating anchoring mode was the most stable form for these dyes for both the *cis* and *trans* isomers. To simulate the dye/TiO_2_ complexes, the initial geometry of the (TiO_2_)_9_ anatase cluster was obtained from the previous study [26], which was large enough to reproduce the electronic and optical properties of the nanocomposites [27].

### 3.2. FT-IR Spectroscopic Analysis

The simulated FT-IR spectra of the two isolated dyes and dye/TiO_2_ complexes in the range of 300–4000 cm^−1^ are shown in Figure S1. IR peaks with high intensity were observed mainly in the regions 1100–1900 cm^−1^ and 3000–3800 cm^−1^ for the *cis* and *trans* isomers of the isolated DMAC dyes. The characteristic peak at 3750 cm^−1^ arose from the stretching vibration of O-H in the carboxyl unit. Compared with the FT-IR spectrum of the dye/TiO_2_ complexes, the O-H stretching vibration was weaker, which indicated that the O-H bond in the carboxyl unit of the DMAC dyes had ruptured. Consequently, the characteristic peak corresponding to the stretching vibration of the Ti-O bond appeared at ~470–490 cm^−1^ (Figure S1a), which indicated the formation of a Ti-O bond and the adsorption of the dye on the TiO_2_ surface. Similarly, in the FT-IR spectra of the isolated DPAC dyes, intense IR peaks were observed in the range of 1000–1900 cm^−1^ and 3000–3800 cm^−1^ for both, the *cis* and *trans* isomers (Figure S1b). The peak at 3752 cm^−1^, originating from the stretching vibration of the O-H bond in the carboxyl unit, disappeared in the FT-IR spectra of the dye/TiO_2_ complexes. A peak appeared at ~487 cm^−1^ in the FT-IR spectra of the dye/TiO_2_ complexes, which was attributed to the stretching vibration of the Ti-O bond. The results indicated that both, the DMAC and DPAC dyes, were adsorbed on the TiO_2_ film in their *cis* and *trans* forms.

### 3.3. Adsorption Energy

**Figure 2:**
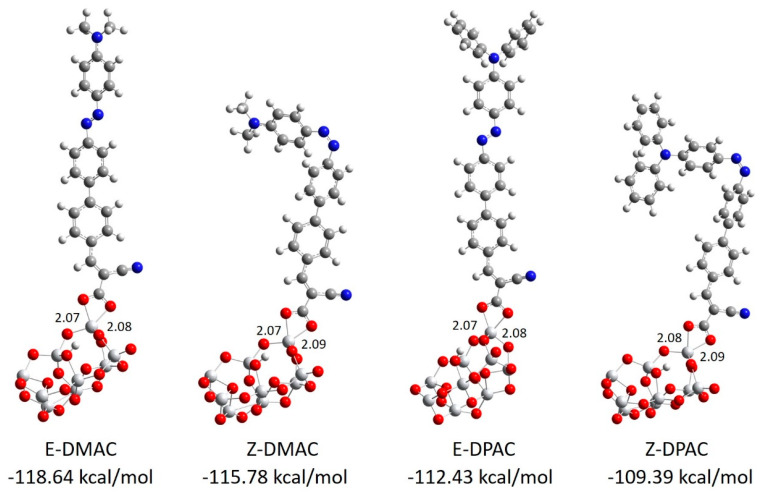
Optimized bidentate chelating mode and adsorption energies of DMAC and DPAC dyes on a (TiO_2_)_9_ anatase cluster calculated at the B3LYP level using the 6-31G(d,p) basis sets for non-metals and LANL2DZ basis sets with ECP for the Ti atom.

The strength of the interaction energy between the dye and the TiO_2_ surface was considered as the adsorption energy, which affected the rate of electron injection. In DSSCs, a high adsorption energy indicates a higher electronic coupling strength between the anchoring group and TiO_2_ surface, which results in higher *J_SC_* as well as electron transfer rate. The optimized structures of the DMAC/(TiO_2_)_9_ and DPAC/(TiO_2_)_9_ complexes are shown in Figure 2. It was evident that the photosensitizers were adsorbed almost perpendicularly onto the TiO_2_ surface with the formation of two Ti-O bonds in the bidentate chelating anchoring mode. The calculated bond distances between the Ti and O atoms of the carboxylic acid of the dyes were in the range of 2.07–2.09 Å, which resulted in a strong interaction between the dyes and the TiO_2_ surface. The adsorption energies of the dyes decreased in the order of *E*-DMAC > *Z*-DMAC > *E*-DPAC > *Z*-DPAC, which implied that the investigated dyes were strongly adsorbed on the TiO_2_ surface. The DMAC dye/TiO_2_ complexes showed a higher adsorption energy than the DPAC dye/TiO_2_ complexes, which increased the electron transfer rate and improved the *J_SC_* and photovoltaic performance of the DMAC dyes.

### 3.4. Structural Analysis

The degree of conjugation of the dyes affects their absorption spectra. Figure 1 shows that the *trans* dyes were fully conjugated as well as extremely coplanar compared to the twisted *cis* structures throughout the donor, π-bridge, and acceptor groups. Because of the strong π-conjugation, the planar *trans* dyes suppressed the rotational disorder and transferred more charge from the donor to the acceptor compared to the distorted *cis* dyes. The angle between the two arene rings of the azo group changed dramatically from 0° to ~78° upon *trans*-to-*cis* photoisomerization of the isolated dyes and dye/TiO_2_ complexes. The dihedral angles between the benzene of the azo moiety and the right part benzene of the π-spacer moiety were ~32.5° owing to the steric hindrance between the hydrogens of the adjacent benzene moieties. The DPAC dyes had a distorted three-dimensional structure with a dihedral angle of ~50° between the phenyl rings owing to the internal steric hindrance among the phenyl rings. The distorted structure was beneficial for inhibiting dye aggregation on the semiconductor. To understand the relationship between the geometric properties and electron-donating strength of the dyes, the selected four bond lengths and the dihedral angle of the azobenzene moiety are summarized in Table 1. The calculated bond lengths were between the bond lengths of single and double bonds (N-C: 1.471 Å, N=C: 1.273 Å, and N=N: 1.247 Å) [28,29,30], which indicated that the charge was delocalized over the entire molecule. Interestingly, the bond length of the azo group (-N=N-), which is an important indicator of ICT in azo dyes, was longer in the *trans* dyes than in the corresponding *cis* dyes for both DMAC and DPAC moieties, while all the C-N bonds of the *trans* dyes were shorter than those of the *cis* dyes. As the electron-donating strength of the donor group increased from DMAC to DPAC dyes, the C-N distances increased; however, the N=N distances decreased in the respective *trans* and *cis* isomers. After binding to the TiO_2_ surface (dye/TiO_2_ complexes), similar trends were observed for both the dyes. The N=N bonds of the *trans* dye/TiO_2_ complexes were longer, while the C-N bonds were shorter than those of the *cis* dye/TiO_2_ complexes. Thus, even with a large displacement from the *trans* to *cis* form, the alternation of bond lengths was observed to be a function of the electron-donating strength. This result suggested that the electron-donating strength affected the geometric properties, which were related to the electronic structures, charge transfer, and optical properties. However, minimal changes were observed in the dihedral angles of the *cis* and *trans* forms of the DMAC and DPAC dyes before and after binding to TiO_2_, indicating that the adsorption on TiO_2_ did not affect the dihedral angles of the azo moiety. It is assumed that the degree of π-conjugation in the azo group could be maintained during the *trans*-*cis* photoisomerization even though the *cis* isomers had a distorted non-planar structure around the azo group.

### 3.5. Cation-to-TiO_2_ Surface Distance

In DSSCs, the undesirable recombination processes are closely related to the contact distance between the cation and semiconductor surface. If the contact distance is small, there is a possibility of electron back-transfer to either the cation or electrolyte during binding to TiO_2_. Because of a smaller cation-to-TiO_2_ distance, the *cis* dyes were expected to exhibit greater recombination while being adsorbed on the TiO_2_ surface, which would lead to lower *J_SC_* and *V_OC_* as compared with those of the *trans* dyes. The contact distance between the cation and TiO_2_ surface is shown in Figure S2. In the case of DMAC dyes (Figure S2a), the cation-to-TiO_2_ distance for the *cis* dye (15.65 Å) was two-thirds of that of the *trans* dye (21.75 Å). A similar trend was observed in the case of DPAC dyes (Figure S2b), where the cation-to-TiO_2_ contact distance for the *cis* dye (13.78 Å) was two-thirds that of the *trans* dye (22.72 Å). This indicated that the *J_SC_* and *V_OC_* of the *trans* isomers were higher than those of the *cis* isomers for both, DMAC and DPAC dyes.

### 3.6. Molecular Orbitals

**Figure 3:**
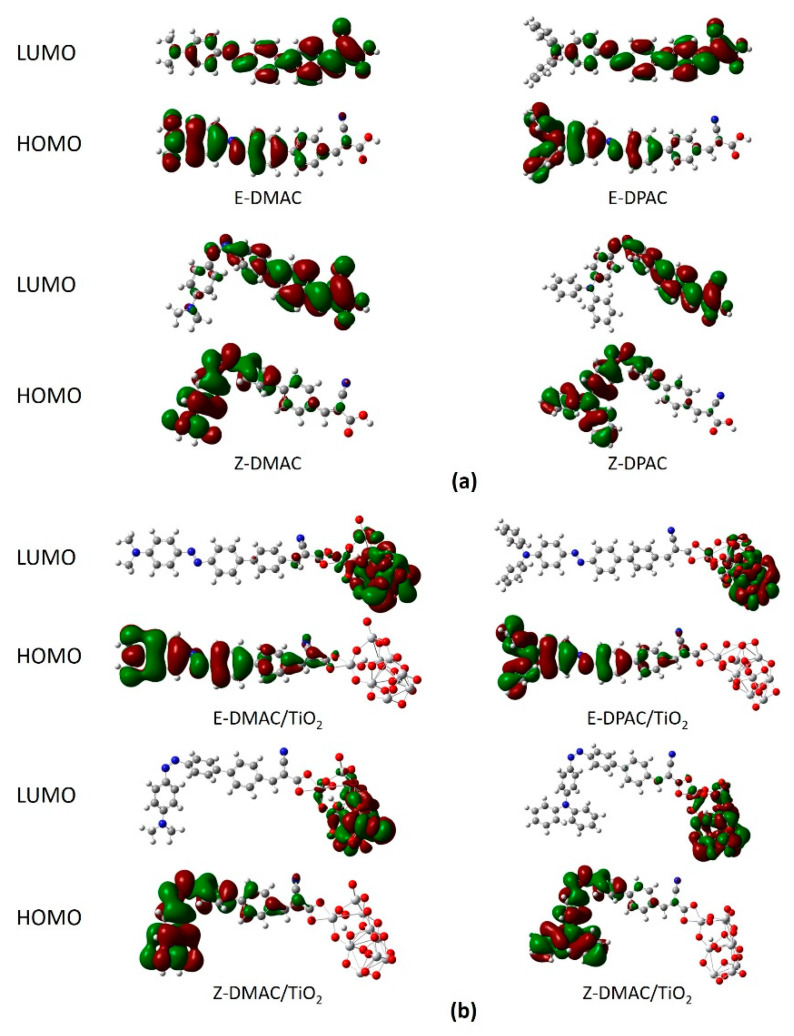
Frontier molecular orbitals of *trans* and *cis* isomers of DMAC and DPAC as (**a**) isolated dyes and (**b**) dye/TiO_2_ complexes.

The frontier molecular orbitals (FMOs) of a molecule can be used to predict its optical and electronic properties. For a better understanding of the electron distribution and the relationship between the electronic structure and electron transition characteristics, the qualitative representation of ICT, i.e., the electron density distributions of the selected FMOs of the two dyes for the *trans* and *cis* isomers are shown in Figure 3.

For both, the DMAC and DPAC dyes (Figure 3a), the electron densities of the HOMOs were extended to the donor up to the azobenzene moiety of the π-spacer, whereas the electron densities of the LUMOs were mainly delocalized along the right part of the π-spacer to the cyanoacrylic acid moiety. The electron distribution of the molecular orbitals confirmed that electron injection occurred from the diarylamine unit (D) to the cyanoacrylic acid unit (A). This was beneficial for the photon-driven ICT process and led to a charge transfer from the donor to the acceptor. ICT is facilitated if the electron density distribution of the HOMO is located near the electron donor, while that of the LUMO is delocalized around an anchoring group, ready for electron injection into the CB of the TiO_2_ semiconductor. Interestingly, the *trans*-*cis* conformation did not affect the HOMO-LUMO electron distribution significantly, which suggested that azobenzene was a good π-spacer for ICT under illumination. Additionally, ICT was maintained even with an evident structural change. Therefore, it was evident that both the *trans* and *cis* forms would serve as a photosensitizer in DSSCs. The electron densities of the FMOs of the dye/TiO_2_ complexes are shown in Figure 3b. The electron densities of the HOMOs for the trans and cis dye/TiO_2_ complexes were distributed from the donor to the π-spacer, similar to the isolated dyes, whereas the electron densities of the LUMOs of the dye/TiO_2_ complexes were almost entirely concentrated on TiO_2_, which indicated that the LUMO located close to the cyanoacrylic acid anchoring group enhanced the orbital overlap with the 3d orbitals of Ti. As a result, the excited electrons were easily injected into TiO_2_ via the anchoring unit, leading to an increase in *J_SC_*. In summary, the study of FMOs suggested that both the dyes showed large ICTs, and consequently, a strong electronic coupling with the TiO_2_ surface.

### 3.7. UV-Visible Spectroscopic Analysis

**Figure 4:**
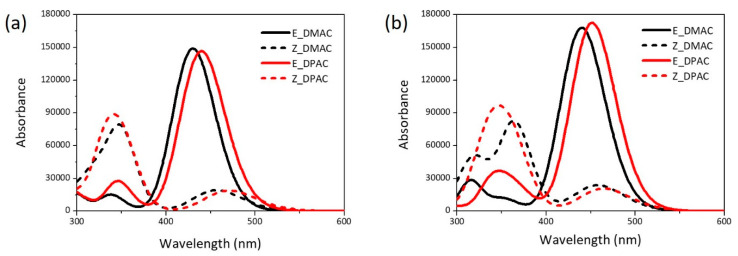
UV-Vis absorption spectra of *cis* and *trans* isomers of DMAC and DPAC as (**a**) isolated dyes and (**b**) dye/TiO_2_ complexes.

The maximum absorption wavelengths (λ*_max_*), oscillator strength (*f*), excited state transition characteristics, nature of the most relevant transitions of the electronic absorption bands, and LHE are summarized in Table 2. The simulated UV-Vis absorption spectra of the DMAC and DPAC dyes in DMF solvent obtained from the TDDFT calculations for the isolated dyes and dye/TiO_2_ complexes are shown in Figure 4. The red and black colors represent the DMAC and DPAC dyes, respectively. The solid and dotted lines represent the *trans* and *cis* dyes, respectively. Both DMAC and DPAC dyes exhibited a broad absorption band and a high molar extinction coefficient, which resulted in the highest sunlight absorption ability. For the isolated dyes (Figure 4a), the *trans* isomers showed a relatively strong absorption at 400–525 nm, with the maximum absorption peaks of the DMAC and DPAC dyes appearing at 430 nm and 440 nm, respectively (Table 2). These strong absorption bands corresponded to the π–π* transition of the FMOs. The absorption ranges of the two dyes were mainly spread over the visible region, thus ensuring effective solar energy usage. Interestingly, two absorption bands were observed for the *cis* dyes. The strong absorption band at ~341–347 nm was possibly due to the π–π* transition, while the weak band at ~457–471 nm could be attributed to the n–π* transition for both *cis* dyes. The spectral difference between the *trans* and *cis* isomers would impart different colors in the DSSC. As the photoirradiation proceeded, the intensity of the *trans* dyes in the 400–500 nm region decreased and that of the *cis* dyes in the 300–400 nm region increased. For the isolated dyes, the major electron transition involved the HOMO, HOMO−1, LUMO, and LUMO+1 orbitals. The change in the electron density between the molecular orbitals (Figure 2) showed that the electron moved from the donor to the acceptor unit, which is an ICT and conducive to a high *J_SC_*. The transition from HOMO/LUMO corresponding to the π–π* transition was the main contributor to the lowest electronic excitation in the *trans* dyes, although transitions from the HOMO/LUMO+1 orbital also contributed to this excitation. In the case of *cis* dyes, the transition from HOMO/LUMO, representing the π–π* transition, contributed to the strong absorption for both, the DMAC and DPAC dyes. The weak absorption by the *cis* dyes was primarily related to HOMO/LUMO+1 of the occupied orbitals corresponding to the n–π* transition, which was due to the presence of unshared electron pairs of the nitrogen atoms. The coplanar structure of the azobenzene unit in the *trans* dyes prevented the n–π* transition, while the n–π* transition in the *cis* dyes resulted from the interaction between the azo bond (N=N) and the π-conjugated system. The transition properties of the dyes adsorbed on the (TiO_2_)_9_ cluster based on the optimized ground-state structures were investigated using the CAM-B3LYP/6-311++G(d,p) method. The isolated dyes and the dye/TiO_2_ complexes exhibited almost similar UV-Vis absorption spectra (Figure 4b). After binding to TiO_2_, the dyes showed a red shift in the maximum absorption wavelengths as compared with those of the isolated dyes. The absorption peaks of the *trans* dye/TiO_2_ complexes showed a red shift of 10–12 nm compared with that of the isolated *trans* dyes, which corresponded mainly to the HOMO/LUMO transition (Table 2). The strong absorption band of the *cis* dye/TiO_2_ complexes, which also corresponded to the HOMO/LUMO transition, showed red shifts of 17 nm (for DMAC dye) and 9 nm (for DPAC dye) compared to those of the isolated dyes, respectively. The red shift of the maximum absorption wavelength of the dye after binding to TiO_2_ could be explained on the basis of the interactions between the electron acceptor group of the dye (–COOH) and the *3d* orbitals of the Ti atom, which resulted in a decrease in the LUMO energies as compared to the isolated dyes. The UV-Vis absorption spectra also revealed the mechanism of photoinjection from the dye to the semiconductor. Compared to the UV-Vis spectrum of the isolated dye, the appearance of a new band in the spectrum of the dye/TiO_2_ complex indicates that it shows a Type II (direct) mechanism [31], whereas the absence of a new band suggests that it exhibits a Type I (indirect) mechanism [32]. As can be seen in Figure 4, both the DMAC and DPAC dyes exhibited a Type I (indirect) injection route during binding to the TiO_2_ surface.

### 3.8. Energy Diagram

**Figure 5:**
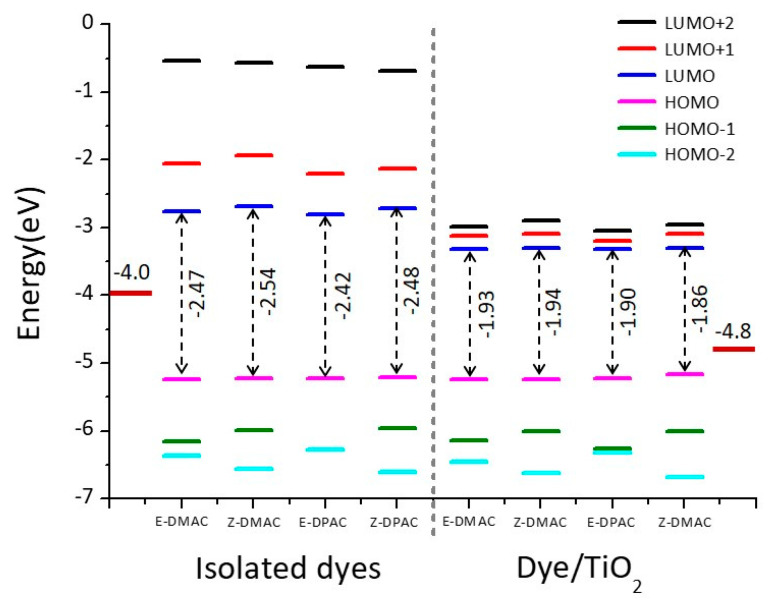
Molecular orbital energy diagrams of *trans* and *cis* isomers of DMAC and DPAC as isolated dyes and dye/TiO_2_ complexes.

To investigate the electronic and transition properties of the dyes, the FMO energy levels from HOMO−2 to LUMO+2 of the isolated dyes and dye/TiO_2_ complexes for both DMAC and DPAC were calculated using the B3LYP/6-311G(d,p) level, and the results are shown in Figure 5.

To design an effective dye, the HOMO and LUMO energy levels of the dyes must be below the redox potential of the I^−^/I^3−^ electrolyte and above the CB of TiO_2_, respectively. The measured HOMO energy levels of the isolated dyes were lower than the redox potential of I^−^/I^3−^ (−4.80 eV) [4], which implied that the oxidized dyes could restore the electrons from the electrolyte. Similarly, the LUMO energy levels of the *trans* and *cis* dyes were above the CB of TiO_2_ (−4.00 eV) [33], which indicated that the designed excited state dyes could quickly and efficiently inject electrons into the TiO_2_ CB. The HOMO-LUMO energy values and their energy gaps are summarized in Table 3. The HOMO energy values of both, the *trans* and *cis* isomers of the DMAC and DPAC dyes, were similar. For LUMO, the DPAC dyes showed a higher energy than the DMAC dyes when comparing their respective isomers. The HOMO-LUMO energy gaps of the *cis* isomers were higher than those of the *trans* isomers owing to the higher LUMO level. The *Z*-DMAC dye exhibited the highest energy difference (2.54 eV), whereas the *E*-DPAC dye exhibited the lowest energy gap (2.42 eV). As the HOMO-LUMO energy gaps of the *trans* dyes were lower than those of the *cis* dyes, they absorbed more light from the visible range and showed a bathochromic shift (Table 2). A higher LUMO level increases the *V_OC_*, thus enhancing the efficiency of the DSSC. Therefore, it is necessary to monitor the enhanced performance of the dye with a higher LUMO energy level. Because of a higher LUMO energy level, the *cis* dyes seemingly had a higher driving force for electron injection compared to the *trans* dyes. However, the *cis* dyes had a larger band gap, which was unfavorable for optical absorption [34]. After binding to the TiO_2_ surface, the FMO energy levels (HOMO−2 to LUMO+2) of the dye/TiO_2_ complexes were calculated to further investigate the electronic coupling between the FMOs and CB of TiO_2_, which are shown in Figure 5. For the dye/TiO_2_ complexes, all the HOMO energy levels were lower than the redox potential of the I^−^/I^3−^ electrolyte and the LUMO energy levels were higher than the CB of TiO_2_, which indicated a strong driving force for electron injection from the dye to the semiconductor as well as a suitable regeneration of the neutral dye. There was almost no change in the energies of the HOMO levels of the dye/TiO_2_ complexes as compared to the isolated dyes. However, the LUMO energy levels remarkably decreased after the dyes adsorbed onto the TiO_2_ surface because of bonding between the semiconductor CB and dye. This implied that the LUMO energy levels of these dyes were strongly coupled with TiO_2_, which is favorable for increasing electron injection into TiO_2_. The HOMO-LUMO energy gap decreased after the dyes adsorbed onto the TiO_2_ surface owing to the relatively low LUMO energy level, which suggested that the adsorption of the dye on the semiconductor surface facilitated the HOMO-LUMO energy level properties crucial for favorable light absorption.

### 3.9. Electrostatic Potential

**Figure 6:**
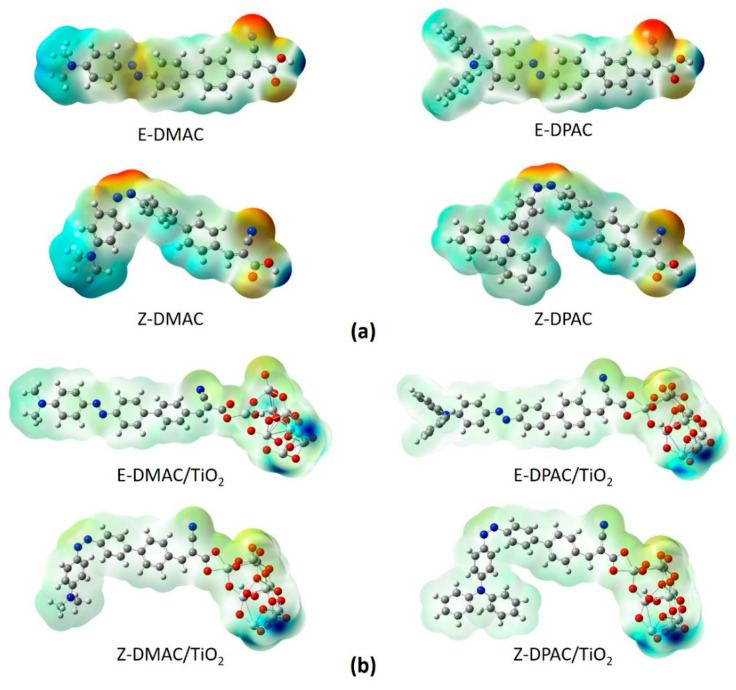
Molecular electrostatic potentials of *cis* and *trans* isomers of DMAC and DPAC as (**a**) isolated dyes and (**b**) dye/TiO_2_ complexes.

To understand the chemical reactions (such as H bonding interactions), the molecular electrostatic potential (MEP), which is closely related to the electron cloud, of the isolated dyes and dye/TiO_2_ complexes, were calculated at the B3LYP/6-311G(d,p) level, and the results are shown in Figure 6. Generally, the MEP is used to describe the nucleophilic and electrophilic reaction sites. The different colors at the surface represent different electrostatic potential values. The red and blue areas of the MEP depict the electrophilic activity corresponding to the electron-rich areas and nucleophilic activity corresponding to the electron-deficient areas, respectively. The electrostatic potential increased in the order: red < orange < yellow < green < blue. The color code of the MEP maps ranged from −0.06 a.u. (deepest red) to 0.06 a.u. (deepest blue). The MEPs of the two isolated dyes (Figure 6a) indicated that the carboxyl H atom in all the dyes had the highest nucleophilic potential. For both dyes, the highest electrophilic potential was exhibited by the N atom of the –CN group in the *trans* structures and the –CN and azo (N=N) groups in the *cis* structures. The H and N atoms represent the strongest attraction and repulsion, respectively. For the dye/TiO_2_ complexes (in Figure 6b), the change was less distinct when the dyes were anchored on the TiO_2_ surface owing to the interactions between the dyes and TiO_2_, which made the dye molecules more neutral in all the regions. However, the highest nucleophilic potential was exhibited mainly by the terminal H of the TiO_2_ cluster, while the highest electrophilic potential was exhibited by the O atoms on the TiO_2_ cluster for both the *cis* and *trans* isomers of the DMAC and DPAC dyes.

### 3.10. Charge Density Difference

**Figure 7:**
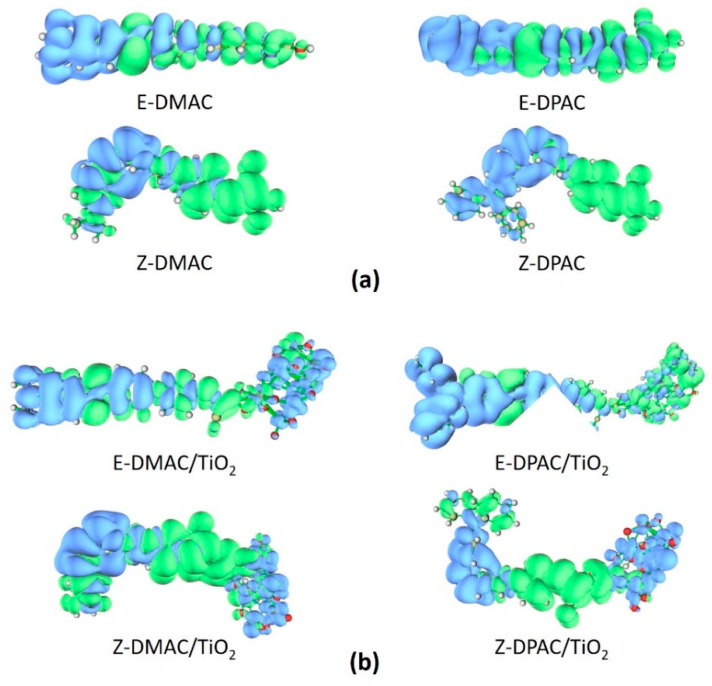
Electron density difference maps for *cis* and *trans* isomers of DMAC and DPAC as (**a**) isolated dyes and (**b**) dye/TiO_2_ complexes.

To investigate the charge transfer properties of the excited state complexes, the charge difference density (CDD) between the excited and ground states of the DMAC and DPAC isolated dyes and dye/TiO_2_ complexes were determined and are shown in Figure 7. The blue and green regions represent the depletion and accumulation of electron density upon excitation, respectively. For the isolated dyes (Figure 7a), the density depletion zones (blue) were mostly located on the donor and π-spacer regions, while the density enhancement segments (green) were mainly delocalized on the acceptor moiety, which was indicative of an ICT transfer during electron transition. The CDD plots of the dye/TiO_2_ complexes (Figure 7b) showed that the density increment region was mostly located on the acceptor moiety, while the density depletion zone was spread over the donor moiety as well as in TiO_2_; this implied that some of the hole and electron densities were delocalized on the dye molecule, while the rest of the electron density was localized on TiO_2_.

### 3.11. NBO Analysis

Based on the optimized structure of the ground state, NBO analysis was performed to further understand the distribution of charge on the overall dye molecules and the electron transfer from the donor to the acceptor through the π-spacer to estimate the extent of ICT. The NBO population charges for the electron donor, π-spacer, and electron acceptor, which are denoted as q^Donor^, q^π-spacer^, and q^Acceptor^, respectively, are summarized in Table 4. The most significant charge variance between the natural charges on the donor and acceptor groups is represented as ∆q^D−A^. The positive NBO values of the donor moiety indicated that they were effective electron-donating units. In contrast, the negative NBO value of the π-spacer suggested that the dye may trap electrons in the π-spacer. The negative charge of the electron acceptor could be a factor leading to electron injection from the excited dye to the TiO_2_ CB. Between the DMAC and DPAC dyes, the former exhibited higher q^donor^ and ∆q^D−A^ values compared to the latter. This indicated that the DMAC dyes could donate more electrons to the anchoring group compared to the DPAC dyes, thus accelerating the ICT. Moreover, the ∆q^D−A^ values of the *trans* dyes were higher than those of the *cis* dyes, suggesting that the ICT ability was sensitive to the conformational changes in the π-spacer. Second order perturbation theory (SOPT) analysis of the Fock matrix on the NBO basis could determine the amount of charge transfer between the different parts of the molecule. Table S1 summarizes the NBO parameters, conjugative interaction energies (∆E^2^) between the π and π* orbitals, energy difference between the interacting NBO and matrix element (E_j_−E_i_), and the off-diagonal element associated with the NBO Fock matrix (F_(i_,_j)_). Carbon atoms (C_1_–C_6_) and nitrogen atoms (N_1_=N_2_) were selected to investigate the electronic delocalization process. A high ∆E^2^ implied more charge transfer from the donor (π) to the acceptor (π*) parts. With increasing donor size, ∆E^2^ increased from the DMAC to DPAC dyes. Furthermore, the ∆E^2^ of the *trans* dyes was noticeably higher than that of the *cis* dyes in the case of π(C_1_=C_2_) to π*(N_1_=N_2_), which indicated that the conformational changes of the dyes also affected the ∆E^2^.

### 3.12. Natural Transition Orbitals and Density of States

The electronic density distributions of the dyes are illustrated in Figure S3 for both the isolated dyes and dye/TiO_2_ complexes determined by natural transition orbital (NTO) analysis. As ICT occurred under light illumination, it was reasonable to analyze the electronic distribution during electronic transition. NTOs can provide detailed information about the excited state transitions apart from the mixed electronic configurations because of multiple excitations among the molecular orbitals. Hole and particle transition orbitals represent the unoccupied and occupied NTOs, respectively. An eigenvalue λ denotes the fraction of the hole-particle pair contribution to the electronic transition. Importantly, the HOMO → LUMO excitation contributed mostly to the S_0_ → S_1_ transition. As shown in Figure S3, the electron density of the hole NTOs was localized on the donor moiety and extended along the π-spacer for the *E*-DMAC and *E*-DPAC dyes, whereas the density was delocalized from the donor to the acceptor moiety for the *cis* dyes. Additionally, the electron density of the particle NTOs was delocalized mainly on the π-spacer to the acceptor moiety for all the dyes. A similar scenario was observed in the case of dye/TiO_2_ complexes for both the DMAC and DPAC dyes. This indicated that photoinduced charge transfer occurs mostly in the *trans* dyes rather than in the *cis* dyes. In addition, the NTO eigenvalues (λ) of the *trans* dyes were higher than those of the *cis* dyes. During visible-light absorption, the electronic transition allowed a net electron transfer from the donor to the acceptor, and subsequently to the TiO_2_ surface. In this regard, the donating capability of the donor was important for charge transfer, as additional noticeable electronic density separation required a stronger donor (Figure S3). The total density of states (TDOS) and partial density of states (PDOS) are represented in Figure S4 for the isolated dyes and dye/TiO_2_ complexes. The vertical dotted line represents the HOMO energy level. For the isolated dyes (Figure S4a), the PDOS of the *p*-orbitals dominated the TDOS of the occupied orbitals, whereas the PDOS of *s*- and *p*-orbitals dominated the TDOS of the unoccupied orbitals for the DMAC and DPAC dyes. In the dye/TiO_2_ complexes (Figure S4b), the PDOS of the *p*-orbitals was the main contributor to the TDOS of the unoccupied orbitals, similar to the isolated dyes. However, for the occupied orbitals, the PDOS of the *p*- and *d*-orbitals dominated the TDOS of the unoccupied orbitals in the dye/TiO_2_ complexes of the two dyes.

### 3.13. Polarizability and Hyperpolarizability

Polarizability and hyperpolarizability characterize the response of a system in an applied electric field. They determine the strength of molecular interactions, such as long-range intermolecular induction and dispersion forces, as well as the cross sections of different scattering and collision processes of the system. Generally, a dye with a higher polarizability strongly interacts with the surrounding species and increases the local concentration of the acceptor species at the TiO_2_ surface, which increases the possibility of the acceptor species penetrating the dye adsorption layer. The total static first hyperpolarizability is expressed as follows [35]:

(1) $$\beta_{tot}=\sqrt{\beta_{x}^{2}+\beta_{y}^{2}+\beta_{z}^{2}}$$

The individual static component in the above equation is calculated from:

(2) $$\beta_{i}=\beta_{iii}+\frac{1}{3}\underset{i\neq j}{\sum }\left(\beta_{ijj}+\beta_{jij}+\beta_{jji}\right)$$

where β_ijk_ (i, j, k = x, y, z) are the tensor components of the total static first hyperpolarizability. Owing to Kleinman symmetry, the following equation is finally obtained:

(3) $$\beta_{tot}=[{{(\beta_{xxx}+\beta_{xyy}+\beta_{xzz}{)}^{2}+{\left(\beta_{yyy}+\beta_{yzz}+\beta_{yxx}\right)}^{2}+(\beta_{zzz}+\beta_{zxx}+\beta_{zyy})}^{2}]}^{1/2}$$

The polarizability and hyperpolarizability of the dyes are shown in Figure S5, and the values are listed in Table S2. The polarizability values of the dyes increased in the order: *E*-DMAC > *E*-DPAC > *Z*-DMAC > *Z*-DPAC. *E*-DMAC exhibited the highest polarizability, which implied that *trans* DMAC was a better dye. Owing to the important application of hyperpolarizability as well as its close relationship with ICT, the first hyperpolarizabilities of the two dyes were also investigated (Figure S5), the results of which are listed in Table S2. The first hyperpolarizabilities of the two dyes were in the order of *Z*-DMAC < *Z*-DPAC < *E*-DMAC < *E*-DPAC. It is noteworthy that all the components of the first hyperpolarizabilities of the two dyes were mainly along β*_xxx_*, which indicated a unidirectional charge transfer from the donor to the acceptor. The β*_total_* values of the *trans* dyes were considerably higher than those of the *cis* dyes, suggesting that the *trans* dyes led to more photoinduced electron transfer in the excited state. Although the first hyperpolarizability of DPAC was higher than that of DMAC, the former prevented electron transfer from the donor to the acceptor because of the non-planar structure of the donor, thereby affecting the effective electron injection from the dye molecule to the CB of the semiconductor.

### 3.14. Other Molecular Properties

Dyes with different dipole moments (Ds) can modify the CB of wide-bandgap semiconductors (e.g., TiO_2_ and ZnO) and affect the nature of the interaction between the dye and the acceptor species. A strong electron-donating ability results in a higher D of the dyes, which can increase the distance between the charge centers, leading to enhanced electron delocalization. The Ds of the isolated dyes and dye/TiO_2_ complexes are listed in Table 3. In the case of isolated dyes, the Ds of the DMAC dyes were higher than those of the DPAC dyes. Moreover, the Ds of the *cis* dyes were higher than those of the *trans* dyes, which increased the bond polarity; thus, the D vectors of the bonds cancelled each other. In the case of dye/TiO_2_ complexes, the Ds of the DMAC dye/TiO_2_ complexes were higher than those of the DPAC dye/TiO_2_ complexes, with the *cis* dye /TiO_2_ complexes showing higher Ds than the *trans* dye/TiO_2_ complexes, similar to the isolated dyes. However, the Ds of the dye/TiO_2_ complexes were significantly higher than those of the isolated dyes, which indicated that after their adsorption on the TiO_2_ surface, the dyes showed greater electron delocalization (Figure 3b). Exciton binding energy (EBE) is another key factor affecting the efficiency of excitonic solar cells and is associated with charge separation in the solar cells. Dyes with high EBEs exhibited the lowest charge separation efficiency. The calculated EBEs of the two dyes are listed in Table 3. In the isolated dyes, the EBE of DMAC was lower than that of DPAC, with the *trans* dyes showing lower EBEs than the *cis* dyes in both the cases, which was a desirable outcome for photo-to-current energy conversion. The dyes with lower EBEs (*trans* dyes) generated current more efficiently from the absorbed light. In the case of the dye/TiO_2_ complexes, the EBEs of the DPAC dyes were higher than those of the DMAC dyes, with the *cis* dye/TiO_2_ complexes showing higher EBEs than the *trans* dye/TiO_2_ complexes. This indicated that the *trans* dye/TiO_2_ complexes had a higher charge separation efficiency than the *cis* dye/TiO_2_ complexes, which was favorable for a better power conversion efficiency (PCE) of DSSCs. The coupling constant (|V_RP_|), a factor that affects the rate of electron injection between the organic dyes and the semiconductor surface, could be derived from the following equation [36]:

|V_RP_| = ∆E*_RP_*/2 (4)

Equation (4) indicates that a high ΔE*_RP_* will result in a high |V_RP_|, which will enhance the electron injection in DSSCs. The ΔE*_RP_* can be estimated as follows [37]:

(5) $$∆E_{RP}=\left[E_{LUMO}^{dye}+2E_{HOMO}^{dye}\right]-\left[E_{LUMO}^{dye}+E_{HOMO}^{dye}+E_{CB}^{TiO_{2}}\right]$$

The experimental value of $E_{CB}^{TiO_{2}}$ was −4.0 eV [33]. The calculated |V_RP_| values of the DMAC and DPAC dyes (listed in Table 3) decreased in the order of *E*-DMAC > *Z*-DMAC > *E*-DPAC > *Z*-DPAC. This trend implied that compared to the DPAC dyes, the DMAC dyes had a higher electron injection rate and the largest number of electrons in the CB, which led to a higher *V_OC_*. A similar phenomenon was observed in the case of the dye/TiO_2_ complexes. *E*-DMAC/TiO_2_ showed the highest |V_RP_|, whereas *Z*-DPAC/TiO_2_ showed the lowest |V_RP_|.

### 3.15. Excited State Lifetime

The efficiency of electron injection to TiO_2_ can be determined by the excited state lifetime. Electron injection from the excited dye to the semiconductor was very fast, which suggested that increasing the concentration of the acceptor on the TiO_2_ surface would increase the possibility of the acceptor species penetrating the adsorbed dye layer, thus leading to electron recombination following a short electron lifetime. This process would minimize the photovoltage and lower the charge collection efficiency, thereby reducing the *J_SC_* and PCE. After electron injection, the dye was in a cationic state until regeneration occurred. It has been reported that the considerable reduction in the electron lifetime in porphyrin-based DSSCs is the main reason for their lower *V_OC_* compared to that of the Ru sensitizer N719 [38]. The longer the excited state lifetime, the longer the dyes remained in the cationic form, which favored charge transfer. The excited state lifetime of the dye was estimated as follows [39]:

τ = 1.499/*f*E^2^ (6)

where E is the excitation energy (cm^−1^) of the different electronic states and *f* is the oscillator strength corresponding to the electronic state. To calculate the excited state lifetimes, the ground-state geometries of the DMAC and DPAC dyes were optimized in their first excited singlet electronic state with the CAM-B3LYP/6-311+G(d,p) level of theory for the isolated dyes and dye/TiO_2_ complexes, considering the lowest excitation energy and the corresponding oscillator strength. The calculated excited state lifetimes of the two dyes are listed in Table 3. In the case of isolated dyes, the excited state lifetimes of the *trans* DPAC dyes were higher than those of their corresponding DMAC dyes and vice versa, respectively, which implied that the DPAC dyes remained stable in the cationic state for a longer time. In the case of the dye/TiO_2_ complexes, interestingly, the opposite scenario was observed. After binding onto the TiO_2_ surface, *trans* DPAC/TiO_2_ exhibited a lower excited state lifetime compared to DMAC/TiO_2_. A similar observation was made in the case of the *cis* dye/TiO_2_ complexes. This indicated that after adsorbing onto the TiO_2_ surface, the DMAC dyes remained in their cationic form for a longer time and allowed a greater charge transfer. This retarded the charge recombination process, which was favorable for a high *V_OC_* and better PCE of DSSCs.

### 3.16. Chemical Reactivity Parameters

Based on the optimized neutral and ionic structures, the chemical reactivity parameters, namely, chemical hardness (η), electron affinity (EA), ionization potential (IP), electrophilicity power (ω), and electron-accepting power (ω^+^), were investigated to further explain the molecular properties of the dyes; these parameters are listed in Table S3. The ω value represents the stabilization energy of the dyes. These ω values of the DMAC dyes were higher than those of the DPAC dyes and increased in the order of *Z*-DPAC < *Z*-DMAC < *E*-DPAC < *E*-DMAC. Thus, the ω values of the *trans* dyes were higher than those of the *cis* dyes, which implied that the former showed a higher energetic stability by attracting the electrons from the environment. The capability to accept an electron from a donor is measured by EA, which can be represented as ω^+^. A higher value of ω^+^ is desirable to achieve a high *J_SC_*. The ω^+^ values of the dyes decreased in the order of *Z*-DPAC < *Z*-DMAC < *E*-DPAC < *E*-DMAC, which indicated that the *trans* DMAC dye had the highest electron-withdrawing ability, and therefore, a higher ability to attract electrons from the acceptor moiety of the dye. Charge injection and balance affect the performance of the DSSC devices. IP and EA represent the energy barriers of both holes and electrons. The IP and EA of the two molecules were calculated by DFT, and these results are listed in Table S3. The IP and EA of the *trans* dyes were respectively lower and higher than those of the *cis* dyes, which promoted the hole-creating and electron-accepting abilities, respectively. Besides, the IP and EA of the DMAC dyes were respectively lower and higher than those of the DPAC dyes. Hence, *E*-DMAC had better hole-creating and electron-accepting abilities. The η value represents the resistance of the dyes to ICT in solar cells. A lower η and higher ω lead to a lower resistance to ICT and a better *J_SC_*, resulting in a higher PCE. Therefore, to increase charge transfer and separation, dyes should have a lower η. The η values of the *trans* dyes were lower than those of the *cis* dyes (Table S3), which suggested that the *trans* dyes would show better efficiency for DSSCs. In addition, the η value of DMAC was lower than that of DPAC; thus, *E*-DMAC exhibited a lower resistance to ICT, leading to a higher *J_SC_*. The chemical reactivity parameters were also measured for the dye/TiO_2_ complexes (Table S3). It was observed that the ω and ω^+^ of the DMAC dye/TiO_2_ complexes were higher than those of the DPAC dye/TiO_2_ complexes for both *trans* and *cis* isomers. Compared to the isolated dyes, the IP and EA of the DMAC dye/TiO_2_ complexes were respectively lower and higher than those of the DPAC dye/TiO_2_ complexes for both *trans* and *cis* dyes. Moreover, the η values of the DMAC dye/TiO_2_ complexes were lower than those of the DPAC dye/TiO_2_ complexes. It was observed that the dye/TiO_2_ complexes showed a similar behavior as that of the isolated dyes. However, the chemical reactivity parameters shown in Table S3 indicate a better performance of the dye/TiO_2_ complexes, in which the dyes are bound to the TiO_2_ surface, compared to the isolated dyes. Based on these chemical reactivity parameters, the DMAC dyes are expected to show better ICT, higher *J_SC_*, and higher PCE for DSSCs.

### 3.17. Factors Affecting Short-Circuit Current Density

In DSSCs, the sunlight-to-electricity conversion efficiency (*n*) of solar cell devices is determined by the *V_OC_*, *J_SC_*, and fill factor (FF), divided by the incident solar power (*P_inc_*) [40]:

(7) $$n=\frac{\left(V_{OC}\right)\left(J_{SC}\right)\left(FF\right)}{P_{inc}}$$

According to Equation (7), the product of *V_OC_* and *J_SC_* should be optimized to improve the efficiency (*n*). In DSSCs, *J_SC_* can be expressed as [40]:

(8) $$J_{SC}=e\int LHE\left(\lambda \right)\phi_{inject}\eta_{collect}d\lambda ,$$

where LHE(λ) is the light-harvesting efficiency at a given wavelength, *ϕ*_*inject*_ is the electron injection efficiency, and *η_collect_* is the charge collection efficiency. All the components of DSSCs are only different for the dyes; hence, *η_collect_* can be assumed a constant. LHE(λ) can be expressed as [41]:

LHE = 1−10^−*f*^, (9)

where *f* represents the oscillator strength of the dyes corresponding to *λ_max_*. Generally, a higher LHE, caused by the higher *f*, increases the light capturing ability and improves the efficiency of the DSSC. Dyes with a small energy gap are beneficial for achieving a red shift in the maximum absorption peak and a relatively high LHE. The LHEs of the isolated dyes and dye/TiO_2_ complexes were calculated and are given in Table 2. The *f* values of the *trans* dyes (Table 2) were higher than that of the *cis* dyes for both, isolated dyes and dye/TiO_2_ complexes, which suggested that the LHE of the *trans* dyes were greater than those of the *cis* dyes. The LHE should be as high as possible to maximize the *J_SC_*. In the case of isolated dyes, the LHE values for the π–π* transition were higher than those for the n–π* transition, which indicates that the former transition was favorable for LHE for both *trans* and *cis* dyes. Moreover, changing the donor moiety in both *trans* and *cis* dyes affected the *f* and LHE, which implied that the LHE was affected by both, the conformational change of the azobenzene bridge structures and the electron-donating strength of the donor group. The LHE of *E*-DMAC was the highest among all the dyes for the isolated dyes and dye/TiO_2_ complexes, which indicated that DMAC could absorb more photons, leading to a higher *J_SC_*. *ϕ_inject_* was related to the injection driving force (∆G_inject_) of the electrons injected from the excited dyes to the semiconductor substrate. According to Preat’s method [42], ∆G_inject_ can be estimated as follows:

∆G_inject_ = E^dye*^ − E_CB_, (10)

where E^dye*^ is the oxidation potential of the dye in the excited state, and E_CB_ is the CB edge of the semiconductor (−4.00 eV) [33]. E^dye*^ can be estimated as follows [43]:

E^dye*^ = E^dye^ − E_0−0_, (11)

where E^dye^ is the redox potential of the ground state of the dye and E_0−0_ is the vertical transition energy associated with *λ*_max_. Note that this relation is only valid if the entropy change during the light absorption process can be neglected. Hence, higher LHE and ∆G_inject_ are beneficial for increasing the *J_SC_*. The ∆G_inject_, E^dye^, E^dye*^, and E_0−0_ for the two dyes were computed and are listed in Table 5. The ∆G_inject_ values of all the dyes were more negative than that of the TiO_2_ CB edge, which indicates that the excited state dyes lie above the TiO_2_ CB, thus promoting electron injection from the excited sensitizer to the TiO_2_ CB. The absolute values of ∆G_inject_ for both the dyes were considerably higher than 0.2 eV; thus, all the dyes showed a sufficient driving force to inject electrons into TiO_2_ [44]. The ∆G_inject_ values for the *trans* dyes were more negative than those of the *cis* dyes, which suggested that the *trans* dyes would exhibit faster electron injection and a higher *J_SC_* compared to the *cis* dyes. However, an excessively high value of ∆G_inject_ can cause energy redundancy, thus leading to a smaller *V_OC_*. The DPAC dyes therefore had a lower *V_OC_* than the DMAC dyes despite having a higher ∆G_inject_. Similar to the isolated dyes, the ∆G_inject_ values of the DMAC dye/TiO_2_ complexes were lower than those of the DPAC dye/TiO_2_ complexes, with the *cis* dye/TiO_2_ complexes showing lower negative ∆G_inject_ values than the *trans* dye/TiO_2_ complexes. This implied that the *trans* dyes would exhibit a faster electron injection. The regeneration efficiency (*η_reg_*), another important factor that affects the *J_SC_*, is determined by the driving force of dye regeneration (∆G_reg_). ∆G_reg_ can be expressed as follows [45]:

∆G_reg_ = E_redox_ − E_dye_ (12)

The *∆G_reg_* of the isolated dyes and dye/TiO_2_ complexes are listed in Table 5. The *∆G_reg_* values of the DMAC dyes were higher than those of the DPAC dyes, which would result in a higher *V_OC_* of the former. Additionally, the *∆G_reg_* values of the *trans* dyes were higher than those of the *cis* dyes. The dye/TiO_2_ complexes showed a similar trend for *∆G_reg_* values as that of the isolated dyes. The *∆G_reg_* values of the DMAC dye/TiO_2_ complexes were higher than those of the DPAC dye/TiO_2_ complexes, whereas the *∆G_reg_* values of the *trans* dyes were higher than those of the *cis* dyes after adsorption onto the TiO_2_ surface.

### 3.18. Factors Affecting Open Circuit Voltage

In DSSCs, the *V_OC_* can be expressed by the following equation [46]:

(13) $$V_{OC}=\frac{E_{CB}+∆E_{CB}}{q}+\frac{k_{b}T}{q}\ln\left(\frac{n_{c}}{N_{CB}}\right)-\frac{E_{redox}}{q},$$

where *q* is the unit charge, *k_b_T* is the thermal energy, *n_c_* is the number of electrons in the CB, *N_CB_* is the density of accessible states in the CB, and *E_redox_* is the electrolyte Fermi level. *∆E_CB_* denotes the shift in E*_CB_* when the dyes are adsorbed on the substrate and is defined as follows [47]:

(14) $$∆E_{CB}=-\frac{\mu_{normal}}{\varepsilon_{0}\varepsilon },$$

where *μ_normal_* is the dipole moment of an individual dye perpendicular to the surface of the semiconductor substrate; *γ* is the surface concentration of dyes; and *ε_0_* and *ε* represent the vacuum permittivity and dielectric permittivity, respectively. Thus, *μ_normal_* is a key factor in determining *V_OC_*. To analyze the relationship with the LUMO, *V_OC_* can be expressed by the following formula [48]:

e*V_OC_* = E_LUMO_ − E_CB_ (15)

To obtain a higher e*V_OC_*, the E_LUMO_ should be as high as possible. The *μ_normal_* and e*V_OC_* values were calculated and are given in Table 5 for the isolated dyes and dye/TiO_2_ complexes. The *μ_normal_* values of the DMAC dyes were higher than those of the DPAC dyes, while the *μ_normal_* values of the *trans* dyes were higher than those of the *cis* dyes for both DMAC and DPAC dyes. The e*V_OC_* values of the two dyes decreased in the order of *Z*-DMAC > *Z*-DPAC > *E*-DMAC > *E*-DPAC, which indicated that the DMAC dyes had higher e*V_OC_* compared to the corresponding isomers of the DPAC dyes. Interestingly, the e*V_OC_* values of the *trans* dyes were lower than those of the *cis* dyes owing to the lower energy level of the LUMO. Although the *cis* dyes showed a higher e*V_OC_*, there was a possibility of electron back-transfer because of the short distance between the cation and TiO_2_ surface in the *cis* structure (Figure S2), which lowered the actual *V_OC_*. After binding onto the TiO_2_ surface, the *μ_normal_* values of the dye/TiO_2_ complexes increased to approximately twice of those of the isolated dyes. Moreover, the *μ_normal_* values of the *trans* dye/TiO_2_ complexes were higher than those of the *cis* dye/TiO_2_ complexes. Because of the increase in the *μ_normal,_* values, the e*V_OC_* values decreased. It was found that the e*V_OC_* values of the dye/TiO_2_ complexes were approximately half those of the isolated dyes. This suggests that after adsorbing onto the TiO_2_ surface, the dyes showed better *μ_normal_* and e*V_OC_* values compared with the isolated dyes, which improved both *V_OC_* and n. However, the dye/TiO_2_ complexes exhibited no distinct change in e*V_OC_* because the LUMO energy levels were very similar for all the dye/TiO_2_ complexes (Table 3). Equation (13) provides only an ideal value for *V_OC_*. However, the real *V_OC_* of a DSSC is generally lower than the theoretical limit because of a backward reaction between the electrons and the redox electrolyte [49]. If the photogenerated electrons are not rapidly transferred to the conducting substrate, the facile recombination of the electrons and oxidized ionic species of the electrolyte will result in a downward photovoltage. Another factor that influences the efficiency of DSSCs is the reorganization energy (λ), which can represent the charge transfer characteristics based on the Marcus electron transfer theory [50]. To enhance the *J_SC_*, the LHE and ϕ_*inject*_ need to be increased, while *λ* needs to be decreased. For fast electron transfer, the λ of the sensitizers must be low. The *λ* can also affect the kinetics of electron injection (*K_inject_*), which can be described as follows [51]:

(16) kinject=Ae[−λ4kBT],

where *A* is a pre-exponential factor that depends on the strength of the electronic coupling between the dye and the surface, *k_B_* is the Boltzmann constant, and *T* is the temperature. The *λ* can be divided into intermolecular and intramolecular recombination energies. The intermolecular recombination energy has no distinct effect on ICT. The energy of the neutral, cationic, and anionic molecules can be used to calculate the reorganization energy. Hence, the intramolecular recombination energy for hole/electron (λ_h_/λ_e_) transfer can be estimated as follows [52]:

(17) $$\lambda_{h}=\left(E_{0}^{+}-E_{+}\right)+\left(E_{+}^{0}-E_{0}\right)=IP-HEP$$

and:

(18) $$\lambda_{e}=\left(E_{0}^{-}-E_{-}\right)+\left(E_{-}^{0}-E_{0}\right)=EA-EEP,$$

where *E_0_* represents the energy of the neutral molecule in the ground state, $E_{0}^{+}$/$E_{0}^{-}$ represents the energy of the cation/anion with the geometry of the neutral molecule, and $E_{+}^{0}$/$E_{-}^{0}$ represents the energy of the neutral molecule with the geometry of the cationic/anionic state. HEP and EEP are the hole and electron extraction potentials, respectively. The λ values of all the dyes were calculated and the results are presented in Table S4. The total reorganization energies, λ_i_ (summation of λ_h_^+^ and λ_e_^−^), of the DMAC dyes were lower than those of the DPAC dyes, which implies that the DMAC dyes would exhibit faster electron transfer, higher *J_SC_*, and consequently, better PCE. Furthermore, the λ*_i_* values of the *trans* dyes were lower than those of the *cis* dyes. Thus, *trans* DMAC dyes were expected to show greater electron injection from the excited states to the TiO_2_ CB owing to their high LHE and low λ*_i_*.

## 4. Conclusions

Two D–π–A metal-free organic dyes featuring an azobenzene spacer were designed, and their structural, electronic, and optical properties were investigated. Moreover, the effects of the substituted donor groups, including the *trans*-*cis*-*trans* conformational change of the azobenzene π-spacer, on the photovoltaic properties were computationally investigated using DFT and TDDFT methods before and after dye adsorption on TiO_2_ for DSSCs. The adsorption energy, FT-IR spectra, cation-to-TiO_2_ distance, FMO, orbital energy gaps, UV-Vis absorption spectra, and other electronic and optical properties of the two dyes, such as MEP, CDD, NBO, polarizability, hyperpolarizability, and NTO were investigated. Additionally, the chemical reactivity parameters of the two dyes, including EA, IP, chemical hardness, electrophilicity power, and electron-donating strength were calculated. Moreover, the key parameters that were closely related to the short-circuit current density and open circuit voltage, including LHE, dipole moment, coupling constant, EBE, excited state lifetime, driving force of electron injection, dye regeneration, total reorganization energy, total dipole moment, and CB edge of the semiconductor were elucidated to determine the primary reasons for the difference in the photovoltaic performance of the two dyes.

The following conclusions were drawn from the calculated results: (i) All the dyes adsorbed well on the TiO_2_ surface, with the DMAC dyes showing a higher electron transfer rate. (ii) The electron-donating strength affected the geometric properties of the dyes, owing to the alteration of the bond lengths. (iii) The DPAC dyes showed a bathochromic shift, compared to the DMAC dyes. (iv) The *cis* dyes accelerated the recombination processes and facilitated electron back-transfer to either the cation or the electrolyte. (v) All the dyes showed ICT, which is essential for charge transfer. (vi) The *cis-trans* conformation did not significantly affect the ICT and the distribution of the FMO electrons, which indicated that azobenzene was a good π-spacer for ICT under illumination. (vii) The dye/TiO_2_ complexes exhibited an indirect injection route because no new absorption bands appeared in the absorption spectra. (viii) After binding onto the TiO_2_ surface, the dyes showed a lower HOMO-LUMO energy gap. (ix) NBO analysis revealed that the *trans* dyes showed a greater charge difference between the donor and acceptor moieties. (x) The lower chemical hardness and IP and the higher electrophilicity power and EA of the *E*-DMAC dye led to a higher *J_SC_*, resulting in excellent PCE. (xi) Because of higher ∆G_inject_, *∆G_regen_*, *τ*, *μ_normal_*, e*V_OC_*, and *∆E_CB_*, and smaller EBE and *λ_I_*, the *E*-DMAC dye exhibited higher *J_SC_* and *V_OC_*. Thus, the DMAC dye was an outstanding candidate for DSSCs. It is expected that molecules with structures similar to that of the DMAC dye can retain photoelectric properties by molecular regulation. However, other properties like the stability (mechanical and thermal) and operability of the dye in actual environments, amount of dye adsorbed on the TiO_2_ surface, and dye aggregation effects, which are not accounted for in this study, must be considered for better understanding of the photoelectrical properties and photovoltaic performance. These findings offer a new approach for the molecular design of dyes with desired absorption colors and will, thus, contribute to the development of novel dyes while providing crucial insights for elucidating the experimental data of DSSCs.

## Figures and Tables

**Table 1:**
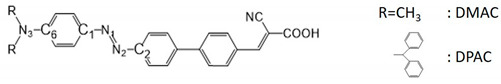
Structural parameters of DMAC and DPAC as isolated dyes and dye/TiO_2_ complexes. A schematic representation of the dye is shown below.

Dye	Bond	Angle (Isolated Dye)	Angle (Dye/TiO_2_)
*E*-DMAC	N_1_=N_2_	1.268	1.272
	C_1_-N_1_=N_2_-C_2_	179.86	178.68
	N_3_=C_6_	1.375	1.368
	C_1_=N_1_	1.397	1.394
	C_2_=N_2_	1.412	1.412
*Z*-DMAC	N_1_=N_2_	1.255	1.261
	C_1_-N_1_=N_2_-C_2_	−11.49	−11.85
	N_3_=C_6_	1.379	1.372
	C_1_=N_1_	1.42	1.412
	C_2_=N_2_	1.425	1.426
*E*-DPAC	N_1_=N_2_	1.267	1.269
	C_1_-N_1_=N_2_-C_2_	179.90	−179.99
	N_3_=C_6_	1.404	1.399
	C_1_=N_1_	1.402	1.400
	C_2_=N_2_	1.414	1.415
*Z*-DPAC	N_1_=N_2_	1.254	1.257
	C_1_-N_1_=N_2_-C_2_	−12.167	−10.94
	N_3_=C_6_	1.407	1.408
	C_1_=N_1_	1.424	1.422
	C_2_=N_2_	1.429	1.431

**Table 2:** Maximum absorption wavelengths (λ_max_), oscillator strengths (*f*), excited state transition characteristics, nature of the transitions for the most relevant transitions of the electronic absorption bands, and light-harvesting efficiencies (LHEs) of the dyes.

Dye	Excited State Character	Transition Assignment (%)	Oscillator Strength, *f*	λ_max_	LHE
*E*-DMAC	π→π*	H-L (66.6%) H-L+1 (32.3%)	2.0486	430	0.9911
*Z*-DMAC	n→π*	H-L+1 (52.9%) H−1-L (21.2%)	0.2647	457	0.4564
	π→π*	H-L (60.2%) H−1-L (18.3%)	0.9926	347	0.8983
*E*-DPAC	π→π*	H-L (65.9%) H-L+1 (26.1%)	1.8015	440	0.9475
*Z*-DPAC	n→π*	H-L+1 (51.4) H−1-L+1 (20.3%)	0.2583	471	0.4483
	π→π*	H-L (64.4%) H−1-L+1 (15.6%)	0.7985	341	0.8411
*E*-DMAC/TiO_2_		H-L (82.6%)	2.3227	440	0.9953
*Z*-DMAC/TiO_2_		H-L+1 (30.1%)	0.3276	458	0.5297
		H-L (53.8%)	1.1195	364	0.9241
*E*-DPAC/TiO_2_		H-L (85.7%)	2.3189	452	0.9951
*Z*-DPAC/TiO_2_		H-L+1 (28.8%)	0.2844	468	0.4805
		H-L (64.3%)	0.8237	350	0.8499

**Table 3:** HOMO and LUMO energy values and energy gaps, excited state lifetimes, dipole moments, exciton binding energies, and coupling constants of the isolated dyes and dye/TiO_2_ complexes.

Dye	HOMO	LUMO	HOMO-LUMO Gap	Ex-State Lifetime, τ	Dipole Moment, D	Exciton Binding Energy, EBE	Coupling Constant, |VRP|
*E*-DMAC	−5.2341	−2.7576	2.477	1.43	11.61	0.41	0.6171
*Z*-DMAC	−5.2276	−2.6858	2.541	1.99	12.20	0.89	0.6138
*E*-DPAC	−5.2270	−2.8066	2.420	1.52	8.67	0.47	0.6135
*Z*-DPAC	−5.2034	−2.7217	2.482	2.57	9.53	0.93	0.6017
*E*-DMAC/TiO_2_	−5.2398	−3.3065	1.936	1.39	22.5	0.88	0.6199
*Z*-DMAC/TiO_2_	−5.2352	−3.2997	1.936	1.95	28.7	1.33	0.6176
*E*-DPAC/TiO_2_	−5.2200	−3.3206	1.900	1.10	21.4	0.98	0.6101
*Z*-DPAC/TiO_2_	−5.1734	−3.3051	1.868	1.74	26.2	1.42	0.5867

**Table 4:** NBO analysis results for metal-free organic dyes in the ground state. Here, q^Donor^, q^π-spacer^, and q^Acceptor^ denote the total amount of natural charges on the donor group, π-spacer, and acceptor group, respectively.

Dyes	q^Donor^	q^π-spacer^	q^Acceptor^	∆q^D-A^
*E*-DMAC	0.3078	−0.1959	−0.1119	0.4197
*Z*-DMAC	0.2523	−0.1395	−0.1127	0.3650
*E*-DPAC	0.2784	−0.1711	−0.1073	0.3858
*Z*-DPAC	0.2090	−0.1010	−0.1079	0.3169

**Table 5:** Electron injection free energy (∆G_inject_), ground (*E^dye^*) and excited (E^dye*^) state oxidation potentials, vertical transition energy (*E_0−0_*), total regeneration energy (*∆G_reg_*), and dipole moment perpendicular to the surface of TiO_2_ (*μ_normal_*) of DMAC and DPAC as isolated dyes and dye/TiO_2_ complexes.

Dye	−*∆G_inject_*	*E_dye_*	E_dye*_	*E_0-0_*	*∆G_reg_*	*μ_normal_*	*eV_OC_*
E-DMAC	−1.649	5.234	2.351	2.883	0.434	12.2	1.243
Z-DMAC	−1.399	5.228	2.602	2.626	0.428	11.6	1.314
E-DPAC	−1.661	5.227	2.339	2.888	0.427	9.5	1.193
Z-DPAC	−1.351	5.203	2.649	2.555	0.403	8.7	1.278
E-DMAC/TiO_2_	−1.578	5.239	2.422	2.817	0.439	24.1	0.694
Z-DMAC/TiO_2_	−1.365	5.235	2.636	2.601	0.435	19.9	0.700
E-DPAC/TiO_2_	−1.663	5.220	2.337	2.883	0.420	21.1	0.680
Z-DPAC/TiO_2_	−1.389	5.173	2.611	2.562	0.373	17.4	0.691

## Supplementary Materials

The following are available online at https://www.mdpi.com/2079-4991/10/5/914/s1, Figure S1: FT-IR spectra of *trans* and *cis* isomers of (**a**) DMAC and (**b**) DPAC as isolated dyes and dye/TiO_2_ complexes, Figure S2: Cation-to-TiO_2_ surface distance of *trans* and *cis* isomers of (**a**) DMAC and (**b**) DPAC dyes, Figure S3: Natural transition orbitals for *trans* and *cis* isomers of DMAC and DPAC as (**a**) isolated dyes and (**b**) dye/TiO_2_ complexes, Figure S4: Total density of states and partial density of states of *trans* and *cis* isomers of DMAC and DPAC as (**a**) isolated dyes and (b) dye/TiO_2_ complexes, Figure S5: (**a**) Polarizability (α*_total_*) and (**b**) hyperpolarizability (β*_total_*) of *trans* and *cis* isomers of isolated DMAC and DPAC dyes., Table S1: Conjugative interaction energies (ΔE^(2)^, in kcal/mol) between the selected π and π* orbitals, Table S2: Polarizability and hyperpolarizability of DMAC and DPAC dyes, Table S3: Electrophilicity index (ω), electron-accepting power (ω^+^), ionization potential (IP), electron affinity (EA), and chemical hardness (η) of DMAC and DPAC as isolated dyes and dye/TiO_2_ complexes, Table S4: Hole extraction potential, electron extraction potential, hole reorganization energy, electron reorganization energy, and total reorganization energy of DMAC and DPAC dyes.
