# Supplementary material for: Theoretical Investigation of Azobenzene-Based Photochromic Dyes for Dye-Sensitized Solar Cells
Source: Nanomaterials (Basel). 2020 May 9;10(5):914. doi: 10.3390/nano10050914 (PMC7279488; doi:10.3390/nano10050914)
Supplement: Supplementary file 1 [file nanomaterials-10-00914-s001.pdf]

nanomaterials

Supplementary Materials

Theoretical Investigation of Azobenzene-Based Photochromic Dyes for Dye-Sensitized Solar Cells

Md Al Mamunur Rashid [1], Dini Hayati [1], Kyungwon Kwak [2,*] and Jongin Hong [1,*]

[1] Department of Chemistry, Chung-Ang University, Seoul 06974, Korea; ndcmamun@korea.ac.kr (M.A.M.R.); dinihayati300194@gmail.com (D.H.)

[2] Center for Molecular Spectroscopy and Dynamics, Institute for Basic Science (IBS) & Department of Chemistry, Korea University, Seoul 02841, Korea

* Correspondence: kkwak@korea.ac.kr (K.K.); hongj@cau.ac.kr (J.H.); Tel.: +82-2-820-5869 (J.H.)

Received: 27 February 2020; Accepted: 16 April 2020; Published: 16 May 2020

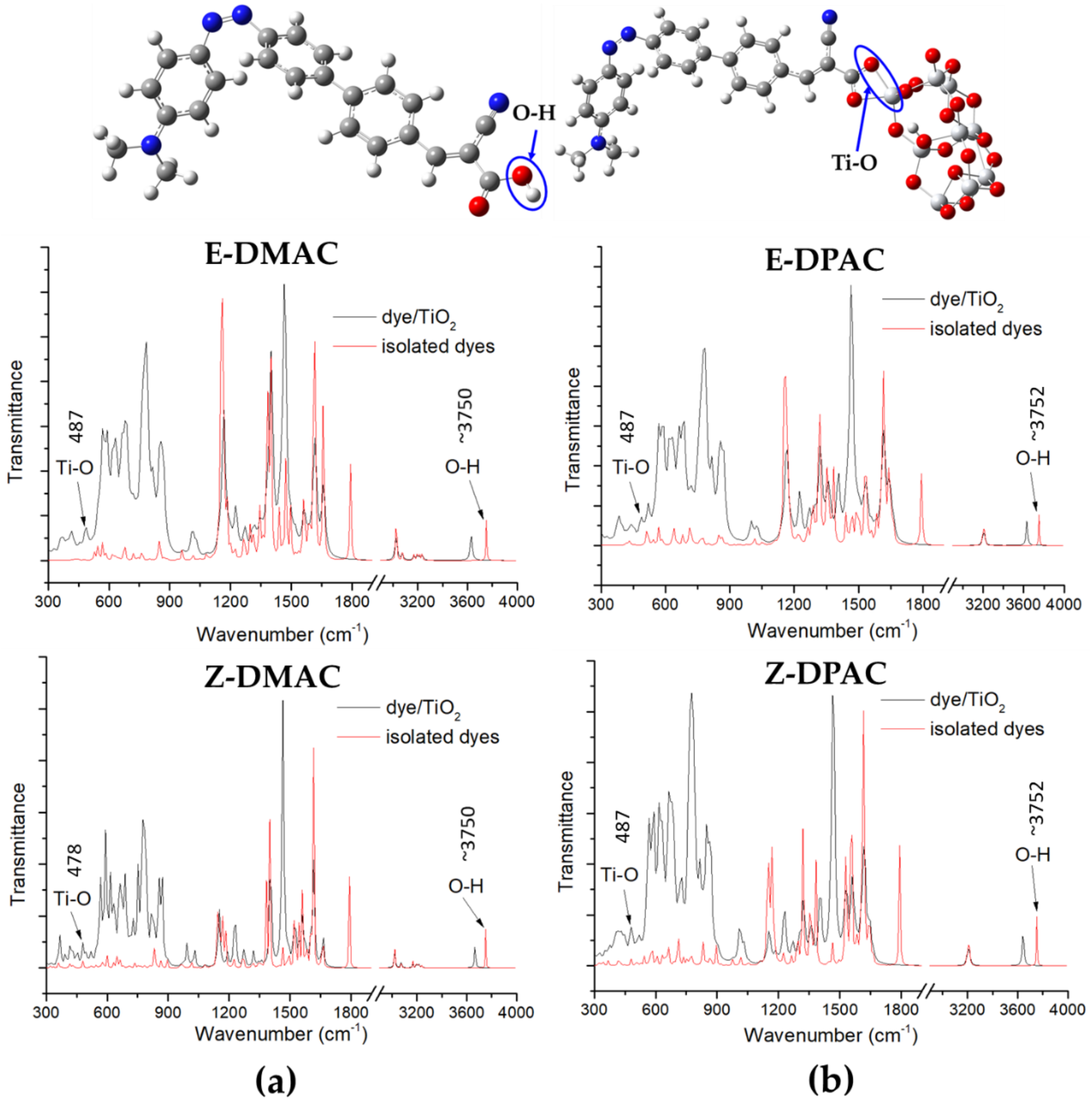

Figure S1. FT-IR spectra of *trans* and *cis* isomers of (**a**) DMAC and (**b**) DPAC as isolated dyes and dye/TiO_2 complexes.

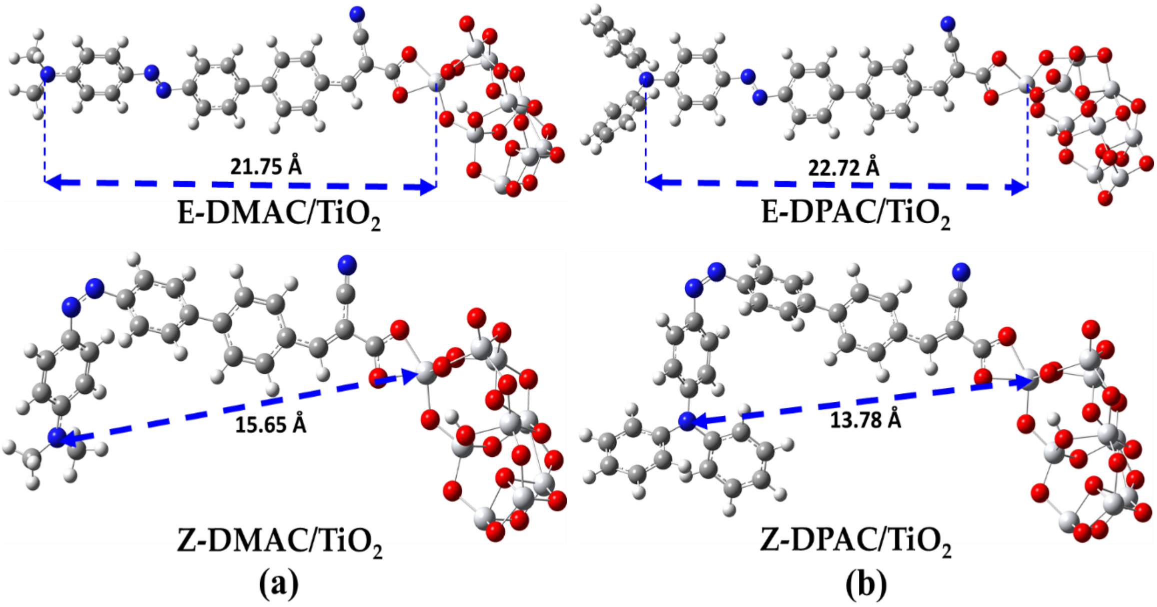

Figure S2. Cation-to-TiO_2 surface distance of *trans* and *cis* isomers of (**a**) DMAC and (**b**) DPAC dyes.

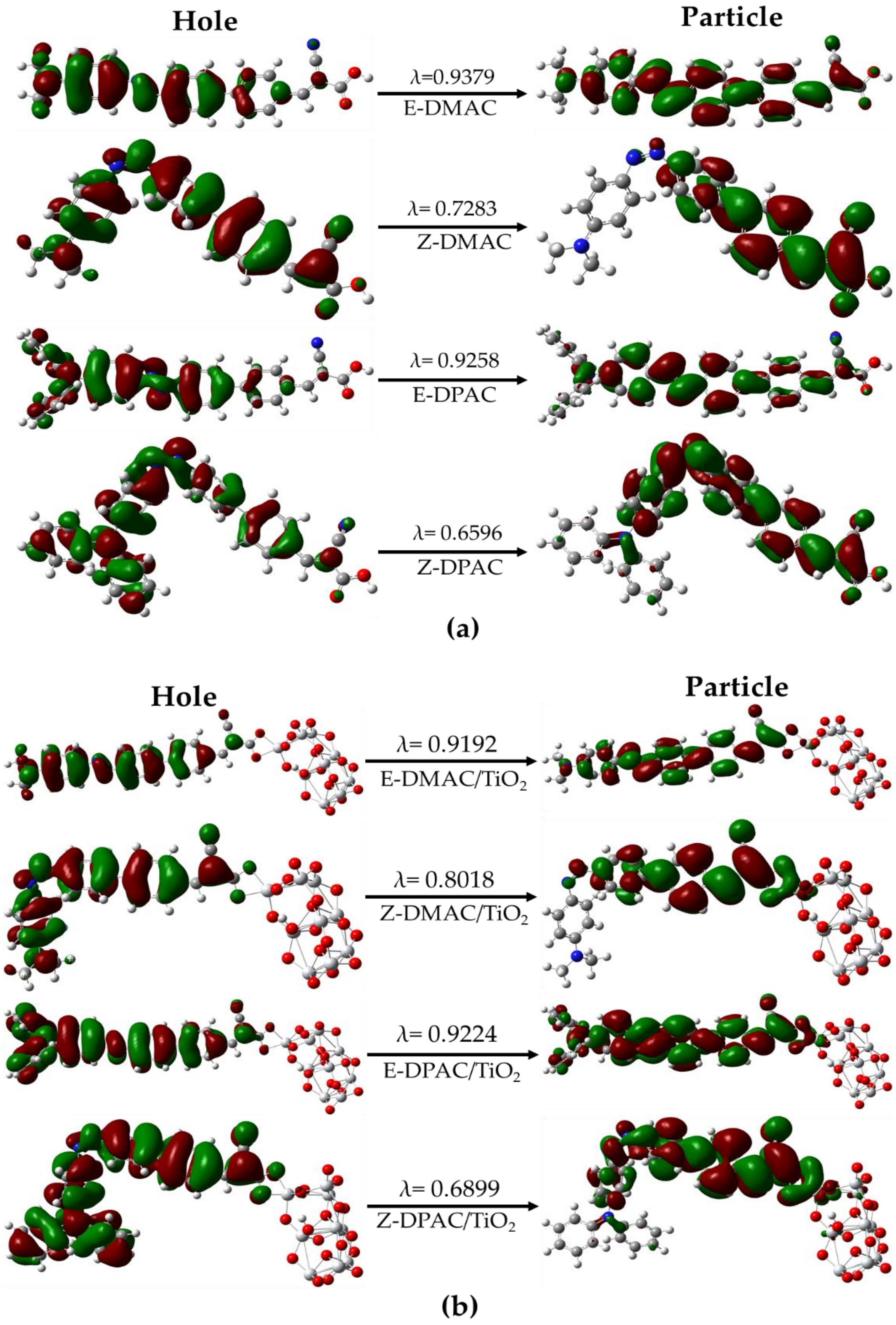

Figure S3. Natural transition orbitals for *trans* and *cis* isomers of DMAC and DPAC as (**a**) isolated dyes and (**b**) dye/TiO_2 complexes.

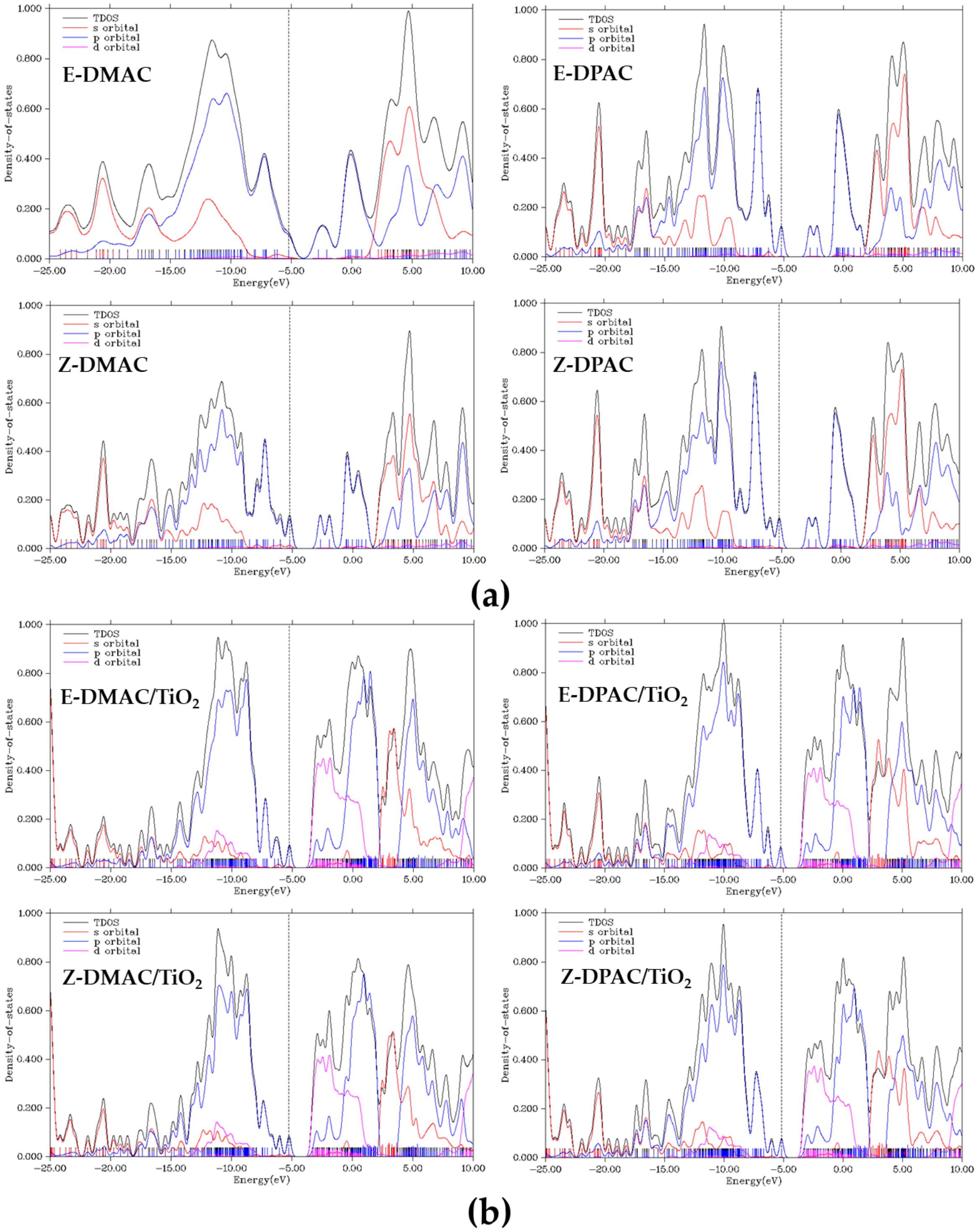

Figure S4. Total density of states and partial density of states of *trans* and *cis* isomers of DMAC and DPAC as (**a**) isolated dyes and (**b**) dye/TiO_2 complexes.

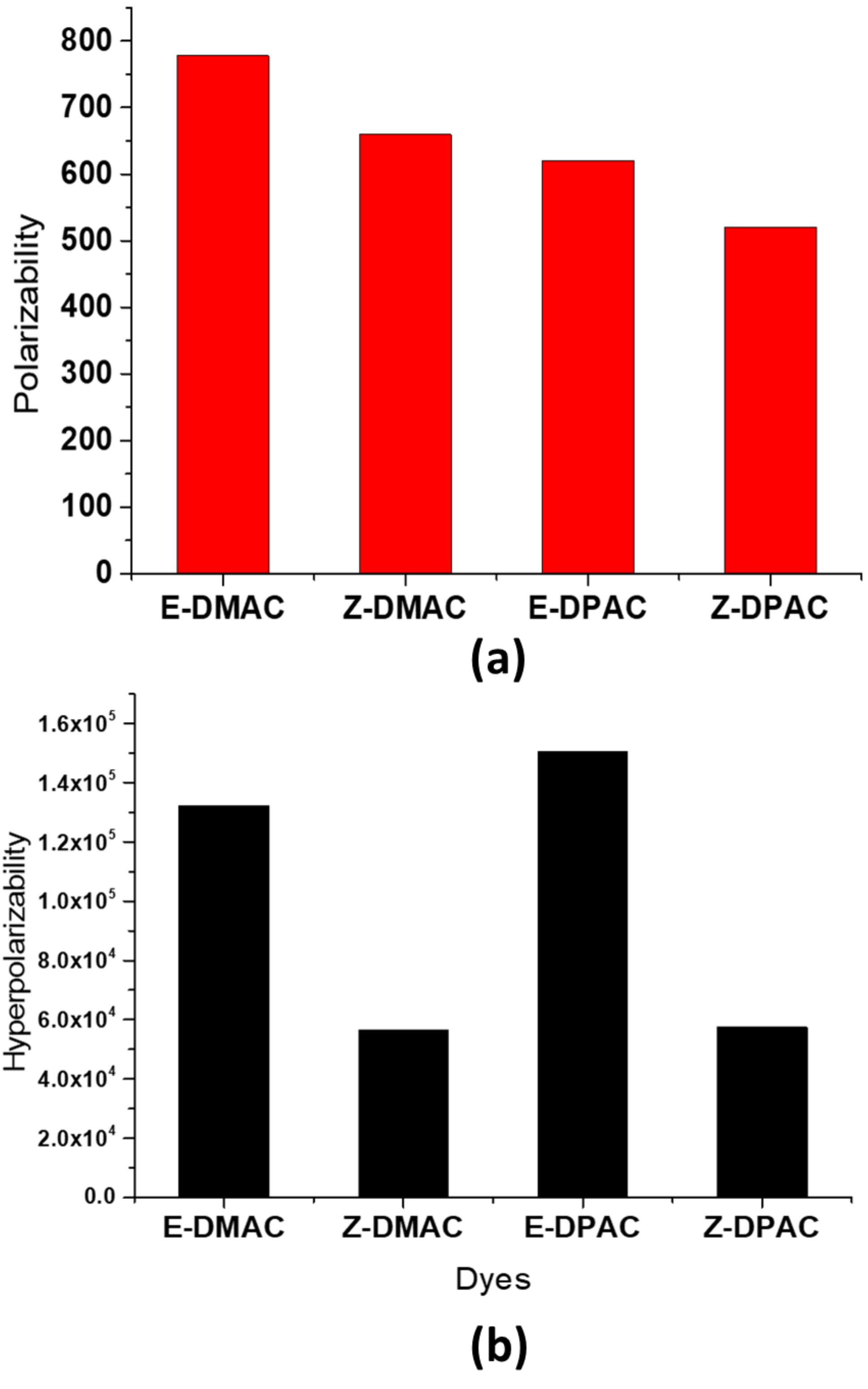

Figure S5. (**a**) Polarizability (α_{total}) and (**b**) hyperpolarizability (β_{total}) of *trans* and *cis* isomers of isolated DMAC and DPAC dyes.

Table S1. Conjugative interaction energies ($\Delta E^{(2)}$, in kcal/mol) between the selected π and π^* orbitals.

Dyes	Donor NBO (i)	Acceptor NBO (j)	$E^{(2)}$ [kcal/mol]	$E_{(j)}$-$E_{(i)}$ [a.u.]	$F_{(i, j)}$ [a.u.]
E-DMAC	$\pi(C_1 - C_2)$	$\pi^*(N_1 = N_2)$	26.34	0.23	0.071
	$\pi(C_3 = C_4)$	$\pi^*(C_5 = C_6)$	23.54	0.28	0.076
E-DPAC	$\pi(C_1 - C_2)$	$\pi^*(N_1 = N_2)$	24.12	0.23	0.069
	$\pi(C_3 = C_4)$	$\pi^*(C_5 = C_6)$	23.31	0.28	0.076
Z-DMAC	$\pi(C_1 - C_2)$	$\pi^*(N_1 = N_2)$	16.14	0.24	0.058
	$\pi(C_3 = C_4)$	$\pi^*(C_5 = C_6)$	23.41	0.28	0.076
Z-DPAC	$\pi(C_1 - C_2)$	$\pi^*(N_1 = N_2)$	15.60	0.24	0.057
	$\pi(C_3 = C_4)$	$\pi^*(C_5 = C_6)$	23.33	0.28	0.076

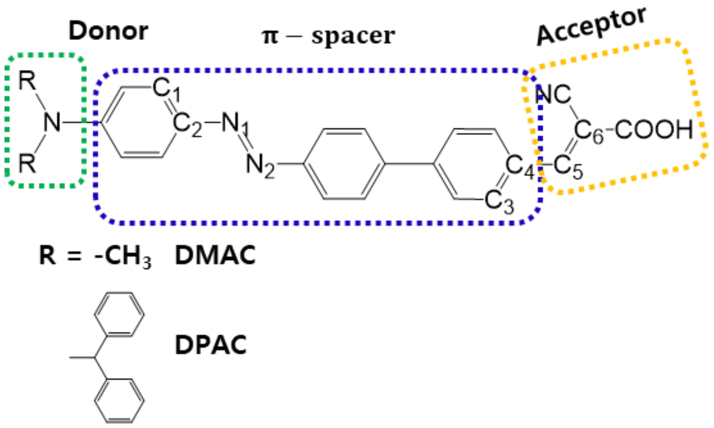

Table S2. Polarizability (α_{total}) and hyperpolarizability (β_{total}) of DMAC and DPAC dyes.

Dyes	α_{xx}	α_{yx}	α_{yy}	α_{zx}	α_{zy}	α_{zz}	$\Delta\alpha$	α_{total}			
E-DMAC	173.39	66.19	442.90	−11.68	257.98	1243.85	1069.22	778.05			
Z-DMAC	563.57	221.50	362.38	−178.94	−62.37	606.72	553.12	659.89			
E-DPAC	515.04	136.46	478.74	−359.75	−129.32	1341.05	1099.04	620.28			
Z-DPAC	612.75	−292.59	581.57	122.30	−141.85	784.93	630.91	520.75			
**Dyes**	β_{xxx}	β_{xxy}	β_{yxy}	β_{yyy}	β_{xxz}	β_{yxz}	β_{yyz}	β_{zxz}	β_{zyz}	β_{zzz}	β_{total}
E-DMAC	2102.2	13532.3	2029.8	−2329.1	3660.5	2009.6	−9763.7	−5034.1	−35331.8	−117354	132189.8
Z-DMAC	1983.7	14718.1	1065.3	7647.2	−1055.6	−836.1	−6375.2	6527.1	6649.6	−1558.2	56377.5
E-DPAC	7209.8	3049.3	6390.6	−2685.4	−18710.5	−8168.6	−2007.4	47345.2	21244.1	−117290	150564.8
Z-DPAC	1356.6	1170.3	−1010.9	8899.8	−8463.5	6234.8	−4961.2	−8101.9	1676.5	−2740.5	57413.1

Table S3. Electrophilicity index (ω), electron-accepting power (ω^+), ionization potential (IP), electron affinity (EA), and chemical hardness (η) of DMAC and DPAC as isolated dyes and dye/TiO_2 complexes.

Dyes	Electrophilicity Power (ω)	Electron Accepting Power (ω^+)	Ionization Potential	Electron Affinity	Hardness (η)
E-DMAC	6.66	4.75	5.10	2.73	1.210
Z-DMAC	6.32	4.13	5.13	2.65	1.240
E-DPAC	6.45	4.67	5.12	2.70	1.238
Z-DPAC	6.15	4.09	5.16	2.61	1.270
E-MAC/TiO_2	9.60	7.96	4.75	3.32	0.934
Z-MAC/TiO_2	9.62	7.48	4.78	3.29	0.949
E-DPAC/TiO_2	9.44	7.77	4.82	3.27	0.966
Z-DPAC/TiO_2	9.40	7.23	4.80	3.22	0.967

Table S4. Hole extraction potential, electron extraction potential, hole reorganization energy, electron reorganization energy, and total reorganization energy of DMAC and DPAC dyes.

Dyes	Hole Extraction Potential	Electron Extraction Potential	Hole-Reorganization Energy (λ_h^+)	Electron-Reorganization Energy (λ_e^-)	Total Reorganization Energy (λ_i)
E-DMAC	4.97	2.39	0.16	0.30	0.46
Z-DMAC	4.83	2.23	0.10	0.37	0.47
E-DPAC	5.08	2.36	0.20	0.34	0.54
Z-DPAC	4.87	2.19	0.14	0.43	0.57
